# Investigation of Healthcare-Associated Infection Risks from Ice: Summary of CDC Consultations 2016-2023

**DOI:** 10.1017/ash.2024.135

**Published:** 2024-09-16

**Authors:** Steven Langerman, Amara Fazal, Matthew Arduino, Kiran Perkins, Christine Yount

**Affiliations:** Centers for Disease Control and Prevention; NCEZID; Division of Healthcare Quality Promotion

## Abstract

**Background:** Nonsterile ice is frequently used in healthcare settings for a wide array of patient care activities and clinical procedures. However, this ice can harbor pathogenic organisms which can threaten patient safety and cause outbreaks. We sought to characterize recent Centers for Disease Control and Prevention (CDC) consultations involving ice leading to healthcare-associated infections (HAIs). **Methods:** We reviewed internal CDC records from the Division of Healthcare Quality Promotion (DHQP) to identify investigations of outbreaks and potential outbreaks involving the use of ice in healthcare facilities. We searched records from January 1, 2016, through November 30, 2023, for keywords related to ice. We excluded consultations in which ice was not thought to be a potential transmission pathway as well as those in which only sterile ice products (e.g., saline slush) were investigated. **Results:** We identified 45 consultations for ice-related investigations, involving a total of 533 patients. Nontuberculous mycobacteria were the most frequently implicated organisms, appearing in 40% (n=18) of investigations. Eighty-four percent (n=38) of investigations occurred in acute care hospitals. The most frequently implicated hospital settings were intensive care units (13%, n=6), operating rooms (13%, n=6), and bronchoscopy suites (13%, n=6). We identified a variety of plausible exposure pathways, including direct ingestion of ice by patients, use of ice during the bronchoscopy procedure, use of nonsterile ice in heater-cooler devices during cardiothoracic surgery, and the use of ice to chill saline for respiratory care. Environmental sampling directly of ice machines was performed in 62% of investigations (n=28) and nonsterile ice from these machines was sampled in 9% of investigations (n=4). Among those investigations in which ice machines were sampled, the organism implicated in the outbreak was isolated in 54% of investigations (n=15). Among those investigations in which ice itself was sampled, the organism implicated in the outbreak was isolated in 75% of investigations (n=3). These organisms included Mycobacterium mucogenicum, Burkholderia multivorans, and Acanthamoeba spp. **Conclusions:** The use of nonsterile ice during clinical care is a potential source of pathogens that cause patient infections and HAI outbreaks. Healthcare personnel should be aware of the risk posed by nonsterile ice and consider avoiding its use, especially when caring for patients who are critically ill or immunocompromised. Healthcare facilities should ensure regular cleaning and disinfection of ice machines to decrease their microbial burden. When HAI outbreaks involving water-associated organisms are identified, nonsterile ice should be considered as a potential mode of transmission.

